# Fluorescent Protein-Tagged Sindbis Virus E2 Glycoprotein Allows Single Particle Analysis of Virus Budding from Live Cells

**DOI:** 10.3390/v7122926

**Published:** 2015-11-27

**Authors:** Joyce Jose, Jinghua Tang, Aaron B. Taylor, Timothy S. Baker, Richard J. Kuhn

**Affiliations:** 1 Department of Biological Sciences, Purdue University, West Lafayette, IN 47907, USA; jjose@purdue.edu; 2 Department of Chemistry and Biochemistry and Division of Biological Sciences, University of California, San Diego, La Jolla, CA 92093, USA; jinghuat@gmail.com (J.T.); tsb@ucsd.edu (T.S.B.); 3 Department of Bindley Bioscience Center, Purdue University, West Lafayette, IN 47907, USA; taylora10@janelia.hhmi.org

**Keywords:** alphavirus, live imaging, virus assembly, filopodia, virus egress

## Abstract

Sindbis virus (SINV) is an enveloped, mosquito-borne alphavirus. Here we generated and characterized a fluorescent protein-tagged (FP-tagged) SINV and found that the presence of the FP-tag (mCherry) affected glycoprotein transport to the plasma membrane whereas the specific infectivity of the virus was not affected. We examined the virions by transmission electron cryo-microscopy and determined the arrangement of the FP-tag on the surface of the virion. The fluorescent proteins are arranged icosahedrally on the virus surface in a stable manner that did not adversely affect receptor binding or fusion functions of E2 and E1, respectively. The delay in surface expression of the viral glycoproteins, as demonstrated by flow cytometry analysis, contributed to a 10-fold reduction in mCherry-E2 virus titer. There is a 1:1 ratio of mCherry to E2 incorporated into the virion, which leads to a strong fluorescence signal and thus facilitates single-particle tracking experiments. We used the FP-tagged virus for high-resolution live-cell imaging to study the spatial and temporal aspects of alphavirus assembly and budding from mammalian cells. These processes were further analyzed by thin section microscopy. The results demonstrate that SINV buds from the plasma membrane of infected cells and is dispersed into the surrounding media or spread to neighboring cells facilitated by its close association with filopodial extensions.

## 1. Introduction

Alphaviruses are arthropod-borne viruses that cause frequent epidemics in humans and other vertebrates. Sindbis virus (SINV) is the type member of the genus *Alphavirus* that replicates in mammalian host and mosquito vector cells. It has a positive-sense, single-stranded RNA genome of 11,703 nucleotides with a cap at the 5′ end and a 3′ poly(A) tail. Nonstructural proteins (nsP1-nsP4) are translated from the 49S genomic RNA, whereas structural proteins capsid (CP), E3, E2, 6K, and El are translated as a polyprotein from a 26S subgenomic RNA [1]. From the structural polyprotein precursor, CP is autoproteolytically cleaved, exposing an N-terminal signal sequence on E3 that translocates the glycoprotein precursor into the endoplasmic reticulum (ER). In the ER lumen, signalase cleavage removes 6K from pE2 (E3-E2) and E1 envelope proteins that are subsequently glycosylated and form heterodimers. These glycoprotein heterodimers trimerize to form glycoprotein spikes that are transported to the plasma membrane (PM) via the secretory pathway [2,3]. Furin cleavage followed by the release of E3 in the late Golgi primes the glycoprotein spikes for subsequent fusogenic activation during cell entry [4]. CP binds genomic RNA in the cytoplasm to form nucleocapsid cores (NCs). Subsequently, virus particles bud from the plasma membrane (PM) where specific interactions between CP and the cytoplasmic domain of E2 (cdE2) drive envelopment and budding of virions [5].

SINV virions are spherical (~70 nm diameter) and contain 240 copies each of CP, E1, and E2 arranged with icosahedral symmetry in a T = 4 lattice [6]. A host-derived lipid bilayer membrane lies sandwiched between the outer glycoprotein shell and the inner nucleocapsid core (NC) that encapsidates the genomic RNA. Virions also contain sub-stoichiometric amounts of the small “6K” and “TF” proteins [7]. There are two types of virus-induced membranous structures found in the infected cells: type I and type II cytopathic vacuoles (CPV-I and CPV-II) [8,9]. CPV-I (0.6 to 2.0 μm diameter) originates from endosomes and lysosomes and contains replication spherules that are the sites of viral RNA synthesis [10]. CPV-II [11] originates from the *trans*-Golgi network ~4 h post-infection (p.i.) [12,13] and contains the E1/E2 glycoproteins with numerous NCs attached to its cytoplasmic face [11,12,14]. Electron tomography studies have revealed that the E1/E2 glycoproteins are arranged in a helical array within CPV-II in a manner that resembles their organization on the viral envelope [2]. CPV-IIs have been proposed earlier to be caused by over-loading of the secretory pathway by the highly expressed viral glycoproteins [12]. Later it was also suggested that CPV-IIs promote the intracellular transport of the glycoproteins from the *trans*-Golgi network to the PM and also the transport of NCs to the site of virus budding at the PM [2]. Subsequently, NC buds through the PM by forming specific interactions with cdE2 [5,15]. The curvature of the preassembled NC, coupled with regularly spaced, strong interactions between the NC and the cytoplasmic domains of the E2 molecules, allows the membrane and embedded glycoprotein spikes to encircle the NC to form enveloped, fully mature virus particles [6].

We and others have described fluorescent fusion proteins including CP [16], E2 [17,18,19,20], and tetra cysteine-labeled structural proteins for virus entry and budding studies [21]. Furthermore, generation of CPV-I in cells infected with Semliki Forest virus has been demonstrated by live-cell imaging coupled with transmission electron microscopy (TEM) [22]. Several imaging studies have utilized fluorescent protein-tagged viruses and subviral particles in single-particle tracking to probe virus entry and assembly. Fluorescently tagged derivatives of Gag-containing human immunodeficiency virus (HIV)-1 virus-like particles were employed to demonstrate assembly, budding, and release of particles from live cells [23]. Similar studies in hepatitis B virus (HBV) have found that the incorporation of only a few fluorescent protein-tagged envelope proteins is sufficient to generate functional, fluorescent virions and subviral particles that enter HBV receptor-positive cells [24]. Furthermore, cryo-electron microscopy (cryoEM) reconstructions have been utilized to determine the organization of fluorescent proteins on purified virus particles. Using cryoEM methods it has been previously shown that green fluorescent protein (GFP)-tagged HBV core particles purified from a bacterial expression system retained icosahedral structure and displayed GFP on its surface [25]. Likewise, a Herpes Simplex Virus 1 GFP-tagged UL17 minor capsid protein was used to determine its location in the capsid vertex-specific component using cryoEM studies [26]. SINV with fluorescent protein labels on the E2 envelope protein has been employed to study virus assembly and budding in living cells. Previous correlative light and electron microscopy studies using fluorescent SINV have provided information about alphavirus budding. Such studies established that glycoprotein E2 is enriched on the PM in localized patches that also contain other viral structural proteins, from which capsid protein interacts with E2 protein for virus budding. This study also suggested that SINV induces reorganization of the PM and cytoskeleton, leading to virus budding from specialized sites [18].

In the current study we characterized the structural stability of an FP-tagged virus and determined the arrangement of mCherry on the virus surface. We provide evidence for the structural stability of the FP-tagged virus and demonstrate that single-particle tracking can be employed to visualize SINV budding from live cells. By employing FP-tagged virus to study virus spread in mammalian cells, we observed that SINV buds from the PM and is associated with filopodial extensions that assist in the dispersal of virions. Comparison of wild-type and budding negative mutant viruses confirmed that fluorescent specks budding from filopodial extensions of mCherry-E2 virus-infected cells are individual virions. By treating infected cells with fusogenic low-pH media, we show that the nascent virions were able to fuse to the PM of filopodial extensions of the infected cells, and we provide evidence for the presence of virions on the outside of these filopodia. This FP-tagged virus can be employed as a tool in high-resolution live and fixed cell imaging coupled with other labeled host proteins and other components to study various aspects of the alphavirus lifecycle.

## 2. Materials and Methods

### 2.1. Cells and Viruses

Baby hamster kidney fibroblast cells (BHK-15) obtained from the American Type Culture Collection (ATCC) were maintained in minimal essential medium [27] supplemented with 10% fetal bovine serum (FBS). All SINV cDNA clones were constructed using standard overlapping PCR mutagenesis from pToto64, a full-length cDNA clone of SINV, as previously described [28]. Viruses were propagated in BHK-15 cells at 37 °C in Minimum Essential Medium (MEM) supplemented with 5% FBS in the presence of 5% CO_2_ unless otherwise noted.

### 2.2. Construction and Characterization of FP-Tagged Virus and Mutants

Sequences that encode mCherry, with additional Ser residues at the N- and C-termini, were cloned after Ser_1_ of E2 replacing E2 Val_2_. Previously characterized cdE2 mutations (_400_YAL_402_/A3 and _416_CC_417_/A2) [5] and an E1 (G91D) fusion loop mutation [29] were generated by overlapping PCR and were cloned into the mCherry-E2 cDNA plasmid using *BssH*II-*BsiW*I restriction sites. The full-length WT and tagged cDNA clones were linearized with *Sac*I, *in vitro* transcribed with SP6 RNA polymerase, and transfected into BHK-15 cells as previously described [5]. Infectious virus produced from the transfected cells was quantified by standard plaque assay using medium over cells collected at 24 h post-electroporation. Plaque phenotypes and virus titers were determined by comparing the mutant with WT Toto64 plaques.

### 2.3. One-Step Growth Curve Analysis

One-step growth analyses were performed as described previously to measure growth kinetics of the mCherry-E2-tagged virus [5]. BHK-15 cells in 35-mm culture dishes were infected with virus at a multiplicity of infection (MOI) of 5 for 1 h at room temperature. Infected cells were washed extensively with MEM and incubated further and culture media were harvested at every hour for 12 h. The amount of infectious virus in the virus supernatant was quantified by titration on BHK cells. All experiments were conducted in triplicate.

### 2.4. Quantitative Real Time RT-PCR

The number of virus particles released at different time points and total RNA molecules in the media over infected cells were determined by qRT-PCR as previously described [30]. RNA was extracted from virus supernatants using the RNeasy kit (Quiagen, Valencia, CA, USA) according to the manufacturer’s instructions. qRT-PCR was performed using the SuperScript III Platinum SYBR Green One-Step qRT-PCR Kit (Invitrogen, Grand Island, NY, USA) with primers 5′-TTCCCTgTgTgCACgTACAT-3′ and 5′- TgAgCCCAACCAgAAgTTTT-3′, which bind to nucleotides 1044–1063 and nucleotides 1130–1149 of the SINV genome, respectively. Amplification reactions were carried out in triplicate in 25 μL sample volumes that contained a 5 μL aliquot of purified viral RNA [5]. Cycling conditions were 4 min at 50 °C and 5 min at 95 °C, followed by 40 cycles of 5 s at 95 °C and 1 min at 60 °C. The number of molecules of viral RNA was determined using a standard curve of the cycle threshold values (CT) determined by qRT-PCR *versus* the number of molecules of *in vitro* transcribed genomic RNA using primers.

### 2.5. Flow Cytometry (FC)

Transport and cell surface expression of E2 in infected cells were assayed using FC and anti-E2 antibody. BHK-15 cells were infected with an MOI of 5. The cells were trypsinized at 6 h, 8 h, and 12 h post-transfection and resuspended in MEM supplemented with 10% FBS. Cells were washed two times with PBS supplemented with 1% FBS and incubated on ice for 1 h with a 1:50 dilution of anti-E2 127 monoclonal antibody. The cells were washed subsequently three times with PBS (1% FBS) and then incubated on ice in the dark for 30 min with a fluorescein-conjugated goat anti-mouse secondary antibody. The cells were washed thrice with PBS (1% FBS) and suspended in 500 μL of PBS and were analyzed on an FC500 flow cytometer (Beckman Coulter, Indianapolis, IN, USA) with the FlowJo software package. Control staining was performed with mock-transfected cells.

### 2.6. Virus Purification, Cryo-Electron Microscopy (cryoEM), and 3D Image Reconstruction

WT and mCherry-E2 viruses were purified according to standard virus purification protocols. Briefly, cell culture supernatants from SINV or mCherry-E2-tagged virus-infected BHK were collected at 12 h p.i. and the media were harvested and clarified by centrifugation for 15 min at 9000 × *g*. Virus particles were pelleted through a 27% sucrose cushion in a Beckman Ti-50.2 rotor at 38,000 rpm for 2 h. The virus pellets were resuspended and loaded onto a 0 to 30% continuous iodixanol gradient, in TNE (50 mM Tris, pH 7.4, 100 mM NaCl, 1 mM EDTA), and centrifuged at 38,000 rpm in a Beckman SW-41 rotor for 2 h. The virus band was extracted by syringe and buffer exchanged using TNE buffer and the presence of the mCherry-E2 tag was confirmed by SDS PAGE analysis.

Small (3 μL) aliquots of the purified mCherry-E2-tagged virus were vitrified for cryoEM via standard, rapid freeze-plunging procedures [31] on Quantifoil holey grids (Quantifoil, Electron Microscopy Sciences , Hatfield, Pennsylvania, USA). Grids were then loaded into a multi-specimen holder and inserted into an FEI Polara microscope and maintained at liquid-nitrogen temperature. Micrographs were recorded on a 4K^2^ Ultrascan CCD (Gatan, Inc., Pleasanton, CA, USA) at a nominal magnification of 51,000× under low-dose conditions (≈15 e/Å^2^) with the microscope operated at 200 keV and the objective lens defocused between 0.9 and 4.7 μm underfocus. Micrographs that exhibited some astigmatism or specimen drift were eliminated from the data set. Individual virus particles were boxed from the remaining 103 micrographs with the program RobEM [32].The Random model computation method [33] was employed to generate an initial 3D map at ~25 Å resolution for the mCherry-E2 insertion mutant. This map was then used as the starting model to initiate orientation and origin determinations for the full set of 9235 particle images using the AUTO3DEM program suite [33] to yield a final 3D map at 11 Å resolution. Graphical representations were generated with RobEM and Chimera [34]. A SINV pseudo-atomic model [6] was used to fit and interpret the mCherry-E2 virus reconstruction. The crystal structure of red fluorescent protein [35] was used to model the densities not accounted for by the virus itself in the mutant. Optimal fitting of the red fluorescent protein model was achieved by rigid body refinement with the *Fit in Map* module of Chimera [34].

### 2.7. Live Cell Imaging

BHK-15 cells were seeded onto a four-chambered borosilicate cover glass (Fischer Scientific, Pittsburgh, PA, USA) and infected with fluorescent virus at an MOI of 50 at 25% confluence. Infected cells were imaged after media were replaced with Opti-MEM I Reduced-Serum Medium (Invitrogen) at specified time points. Live imaging-compatible stains were obtained from Invitrogen/Molecular Probes. These included Hoechst stain (nucleus) and BODIPY FL C5 ceramide (Golgi stain) and were used according to the manufacturer’s instructions in conjunction with mCherry-E2 virus. Fluorescent images were acquired at indicated temperatures using Nikon A1R confocal microscope (Nikon, Melville, NY, USA ) with 60×, 1.4 numerical aperture (NA) lens) using NIS Elements software (Nikon, Melville, NY, USA). Live imaging for 10–30 min periods was conducted using a heated 60× oil immersion objective (1.4 NA) in a live imaging chamber (Tokai Hit, Fujinomiya, Shizuoka Prefecture, Japan) supplied with 5% CO_2_ at 37 °C. The lasers and emission band passes used for imaging were as follows: blue, excitation: 405 nm, emission: 425–475 nm; green, excitation: 488 nm, emission: 500–550 nm; red, excitation: 561 nm, emission: 570–620 nm. Differential interference contrast images were collected from transmitted light along with fluorescent images for colocalization of viral proteins in the cellular organelles. NIS-Elements software was used for image acquisition and analysis. For generating videos, live images were collected at frame rates ranging from 0.8 to 1 frames per second (fps) for a time scale of 1–30 min, and time-lapse videos were generated from the acquired images at a frame rate of 5–7 fps using ImageJ (NIH Bethesda, Maryland, USA). To compare the size and fluorescent properties of purified mCherry-E2 virus, purified virus was mixed with 0.1 μm diameter fluorescent microspheres (TetraSpeck Beads, Invitrogen) and imaged on a cover glass using a Nikon A1R system with a 60x oil immersion objective (1.4 NA).

### 2.8. Immunofluorescence (IF) Analysis

IF analyses were performed on BHK-15 cells grown on glass coverslips. Primary antibodies used in the experiments were Golgi-specific rabbit polyclonal anti-Giantin (Abcam, Cambridge, MA, USA), SINV-specific rabbit polyclonal anti-E1, anti-CP and mouse monoclonal anti-E2. Cells were fixed using 3.7% paraformaldehyde for 15 min at room temperature and permeabilized using 0.1% Triton × 100 in phosphate-buffered saline (PBS) for 5 min. The secondary antibodies used were fluorescein isothiocyanate (FITC) or tetramethyl rhodamine (TRITC)-conjugated goat anti-rabbit and goat anti-mouse antibodies [36] in PBS with 10 mg/mL bovine serum albumin. Nuclei were stained using Hoechst stain (Invitrogen) according to the manufacturer’s instructions. Images were acquired using a Nikon A1R-MP confocal microscope at room temperature with a 60× oil objective and 1.4 NA. Images were processed using the NIS Elements software (Nikon) and the brightness and contrast were adjusted using nonlinear lookup tables.

### 2.9. Thin-Section Transmission Electron Microscopy (TEM)

BHK-15 cells infected with wild-type or mCherry-E2-tagged SINV at an MOI 5 were fixed at 6 or 12 h p.i. Cells were fixed for three days in 2.5% glutaraldehyde in 0.1 M sodium cacodylate buffer, embedded in 2% agarose, post-fixed for 90 min in buffered 1% osmium tetroxide containing 0.8% potassium ferricyanide, and stained for 45 min in 2% uranyl acetate. They were then dehydrated with a graded series of ethanol, transferred into propylene oxide and embedded in EMbed-812 resin. Thin sections were cut on a Reichert-Jung Ultracut E ultramicrotome and stained with 2% uranyl acetate and lead citrate [37]. Images were acquired in an FEI Tecnai G^2^ 20 electron microscope equipped with a LaB_6_ source and operated at 100 keV (Life Science Microscopy Facility, Purdue University, West Lafayette, IN, USA).

## 3. Results

### 3.1. Construction and Characterization of mCherry-E2 SINV

**Figure 1:**
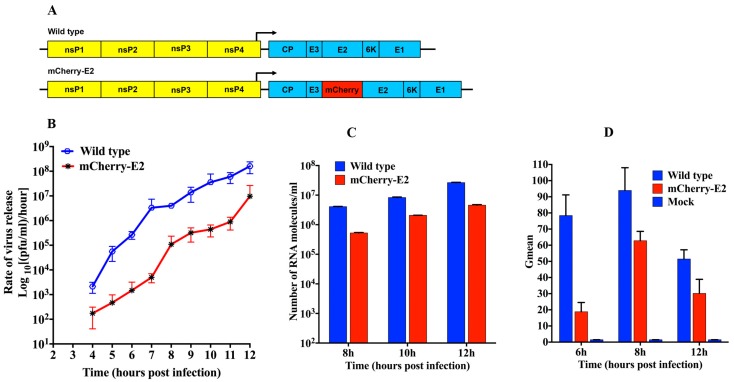
Construction and characterization of mCherry-tagged virus. (**A**) Schematic of wild-type (WT) and mCherry-E2 Sindbis virus (SINV) complementary DNA (cDNA) clones; (**B**) One-step growth curve analysis of WT and mCherry-E2 virus released from Baby Hamster Kidney fibroblast (BHK) cells. BHK cells were infected with WT or mCherry-E2 viruses at a multiplicity of infection (MOI) of 5 and media were changed every hour for 12 h and the rate of virus release (plaque-forming unit (pfu) per ml per hour) was determined using standard plaque assays; (**C**) Quantitation of the number of viral RNA molecules (corresponding to virus particles released into the media) for WT and mCherry-E2 mutant viruses at 8, 10, and 12 h. Total number of genome RNA molecules was determined by quantitative real-time reverse transcription PCR (qRT-PCR) using a standard curve of known amount of *in vitro* transcribed SINV RNA molecules; (**D**) Determination of E2 surface expression in BHK cells infected with WT or mCherry-E2 virus by flow cytometry using anti-E2 monoclonal antibody at 6, 8, and 12 h post-infection (p.i). Y-axis is represented by Gmean, which corresponds to the geometric mean of the fluorescence data calculated by averaging the log of the fluorescence and the scale value of that average in fluorescence units.

FP-tagged SINV constructs have proved to be useful tools to detect replication complexes (RCs) from infected cells and to study virus entry and budding [18,38]. The schematic of the mCherry-E2 construct generated for this study is shown in Figure 1A.

The N-terminus of E2 is known to tolerate insertions [39]. Hence, we cloned the sequence that encodes mCherry into the second residue following the furin cleavage site between E3 and E2 as previously described by us [20] and others [18,19,20]. We extensively characterized this mCherry-E2 virus and compared it with wild-type (WT) SINV. One-step growth kinetic analyses of the mCherry-E2 virus were performed and replication was found to be reduced by a one log equivalent in virus yield compared to the WT virus (Figure 1B). To determine whether the lower number of virus plaque-forming unit (pfu) observed in the growth kinetic analysis was caused by a reduced specific infectivity, quantitative real-time reverse transcription PCR (qRT-PCR) analysis of the number of RNA molecules released into the media was performed (Figure 1C). This analysis showed a consistent reduction in the number of RNA molecules released into the media compared to the WT virus. Based on the calculated particle-to-pfu ratio at each time point (data not shown), the specific infectivity of the mCherry-E2 virus was found to be comparable to that of WT SINV. To further characterize the defect(s) of the mCherry-E2 virus, we analyzed infected BHK cells by flow cytometry (FC) using monoclonal anti-E2 antibody (Figure 1D) and determined the surface expression of glycoprotein spikes at 6, 8, and 12 h p.i. This revealed that the viral glycoproteins were transported to the PM more slowly for the mCherry-E2 virus compared to the WT virus. Also, fewer glycoproteins accumulated at the PM in the FP-tagged virus compared to the WT virus. Indeed, when glycoprotein transport to the PM reached a maximum, the level of mCherry-E2 only reached 65% of the WT E2. This defect in surface expression is likely a consequence of slower folding of the glycoproteins caused by the mCherry insertion, thus resulting in reduced virus production but no assembly defects.

**Figure 2:**
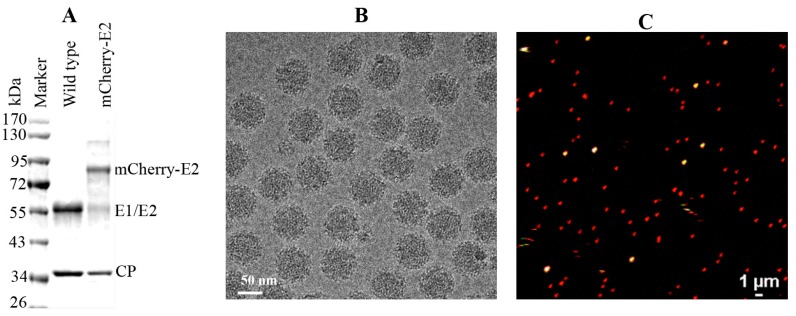
(**A**) Sodium dodecyl sulfate polyacrylamide gel electrophoresis (SDS-PAGE) analysis of purified WT and mCherry-E2 virus showing the size difference of mCherry protein tagged to the E2 and WT E2 protein; (**B**) Cryo-electron microscopy (CryoEM) of purified mCherry-E2 virus exhibiting uniform spherical morphology; (**C**) Confocal merged image of purified mCherry-E2 virus mixed with tetra speck beads (100 nm size fluorescent microspheres fluoresce in green, red, and blue channels, seen as white dots when merged) demonstrating that the individual virions can be observed by confocal live imaging.

As a component of the spike, the mCherry-E2 tag was packaged successfully into particles. To confirm that mCherry was incorporated into particles, we purified the mCherry-E2 virus and compared it with the WT virus by sodium dodecyl sulfate polyacrylamide gel electrophoresis (SDS-PAGE) analysis (Figure 2A). As predicted, the mCherry-E2 protein was larger than the WT E2. Even though the mCherry represents a large insertion in the E2 protein, the tagged virus particles proved to be comparable in size and shape to WT SINV in micrographs of vitrified (Figure 2B) as well as negatively stained samples (data not shown). Next we tested the fluorescence of virus particles after red, green, and blue 100 nm TetraSpeck fluorescent microspheres were mixed with purified mCherry-E2 virus and imaged by confocal microscopy. This test confirmed that the mCherry-E2 virus was suitable for single-particle tracking experiments (Figure 2C).

### 3.2. mCherry-E2 SINV Assembles in a Manner Similar to that of WT Virus

**Figure 3:**
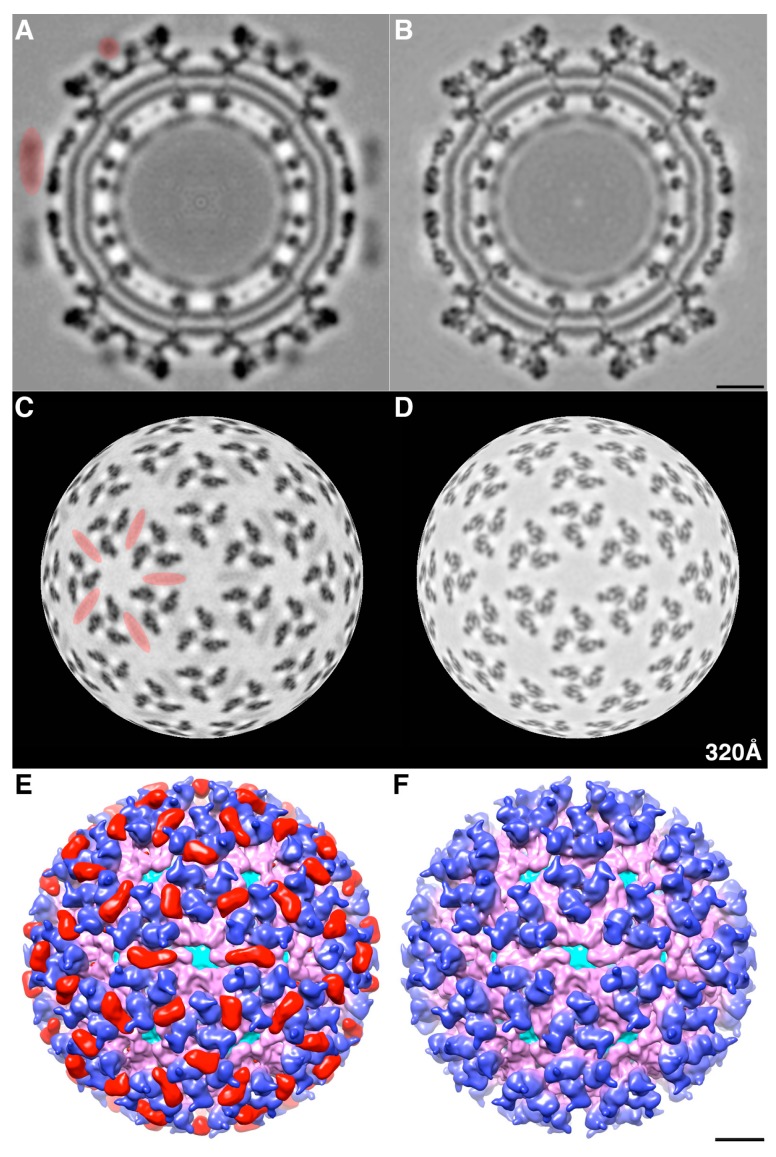
Comparison of mCherry-E2 and WT virus cryoEM structures: (**A**) Central section of mCherry-E2 virus showing extra densities marked in red compared to WT virus; (**B**) Radial projection views at radius of 320 Å show extra densities on the surface of the mCherry-E2 virus (Panel **C**, highlighted in red) compared to WT virus (**D**). Surface view, color-cued by radius (from cyan to pink to blue with increasing radius) of the mCherry-E2 virus (**E**) reveals extra densities (colored in red) compared to the WT virus (**F**). The scale bars represent 100 Å.

The addition of mCherry (236 residues) at the N-terminus of E2 produced virus particles of similar size and shape to native virions. Images of vitrified, FP-tagged particles (Figure 2B) that revealed additional density features at the peripheries of cryoEM reconstructions compared with native virions correlated directly with the presence of mCherry (Figure 3).

**Figure 4:**
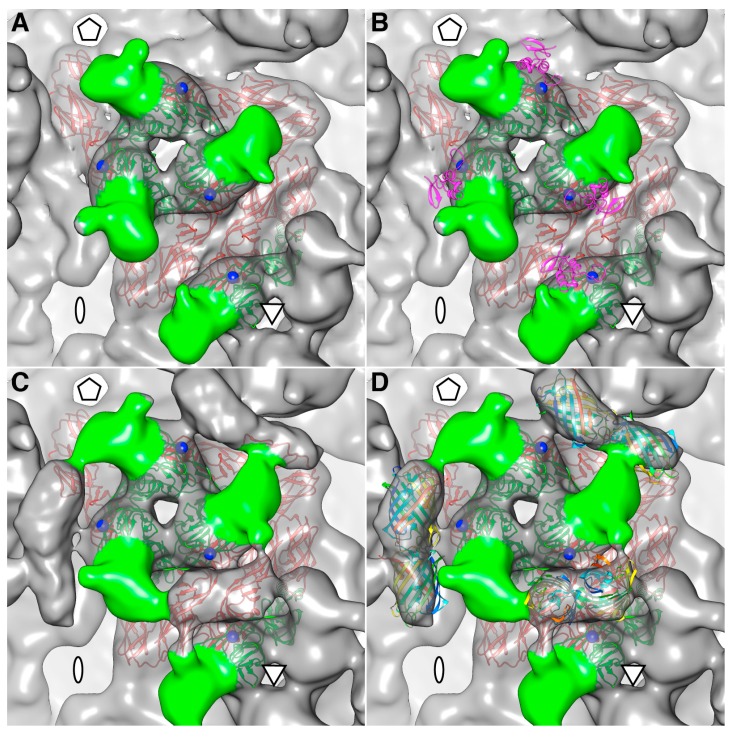
Comparison of mCherry-E2 and WT SINV spike structures. The asymmetric unit of the icosahedral reconstruction is shown with five-, two-, and three-fold axes labeled with a pentagon, oval, and triangle, respectively. The E1 and E2 glycoprotein models in ribbon structures are colored red and green, respectively. The N-terminal residue in each E2 is marked with a blue sphere and the E2 receptor-binding domain represented by the surface structure is colored green. (**A**) WT virus spike; (**B**) WT spike showing the location of E3 on the glycoprotein spike by adding a ribbon structure of E3 (magenta) to the glycoprotein model to show its potential location on the virus surface where the mCherry tag is cloned; (**C**) mCherry-E2 virus spike with extra mCherry densities between the spikes next to the N-termini of E2; (**D**) mCherry-E2 virus spike fitted with the red fluorescent protein (RFP) dimer (rainbow-colored ribbon structure from N- to C-termini) between two neighboring E2 subunits across the spikes.

FP-tagged particles exhibited additional morphological features on the outer surface compared with the native virion, which we attribute to the mCherry moiety (Figure 3A,C,E). The observation that moderate resolution three-dimensional (3D) cryo-reconstructions could be computed from images of mCherry-E2-tagged particles proved that they were uniform and stable analogous to native SINV (Figure 3E,F and Figure 4A,C). In native SINV, after pE2 is cleaved by furin, the N-terminus of E2 is located at the surface of E2 [40]. Because the mCherry tag occurs at the N-terminus of E2 and coincides with the location of the uncleaved E3, we generated an E3 model for comparison (Figure 4B). The E3 model was built by fitting the E3 structure of Chikungunya virus (CHIKV) [40] into the cryoEM reconstruction of an uncleaved E3 mutant reconstruction. The location of mCherry density near the tip of the glycoprotein spike (Figure 4C) was consistent with the position of the E2 N-terminus and with E3. The mCherry tag is oriented such that it splays away from the E2 receptor binding domain and the E1 fusion peptide. Hence, its presence does not interfere with the infectivity of the mutant virus. However, given that the mCherry tag wedges between adjacent spikes (Figure 3E and Figure 4D), some small conformational changes occur in the spikes and in the NC protein shell (Figure 3A,B).

### 3.3. SINV Budding Observed in Live Cell Imaging

**Figure 5:**
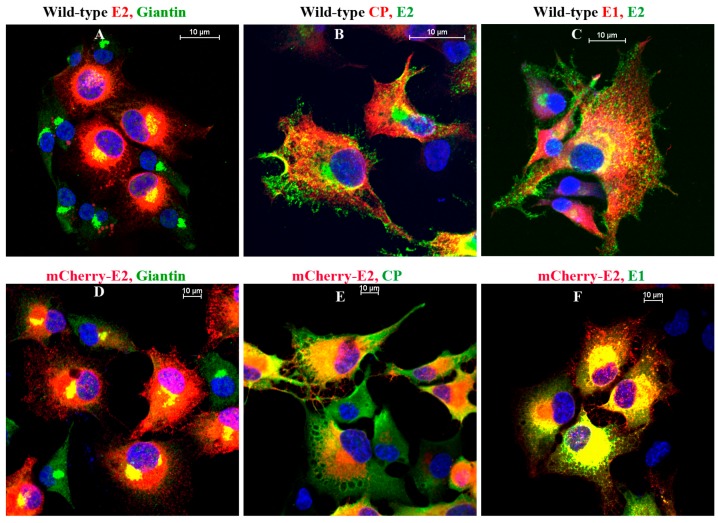
Immunofluorescence (IF) analysis of WT (**A**–**C**) and mCherry-E2 virus-infected BHK cells (**D**–**F**) showing the distribution of viral proteins. Cells infected with WT or mCherry-E2 viruses were subjected to IF analysis at 6 h p.i. with antibodies against rabbit polyclonal Giantin (Golgi), rabbit polyclonal anti-CP, rabbit polyclonal anti-E1, and mouse monoclonal anti-E2 primary antibodies and labeled with fluorescein isothiocyanate (FITC)- or tetramethylrhodamine (TRITC)-labeled goat anti-rabbit and goat anti-mouse secondary antibodies.

**Figure 6:**
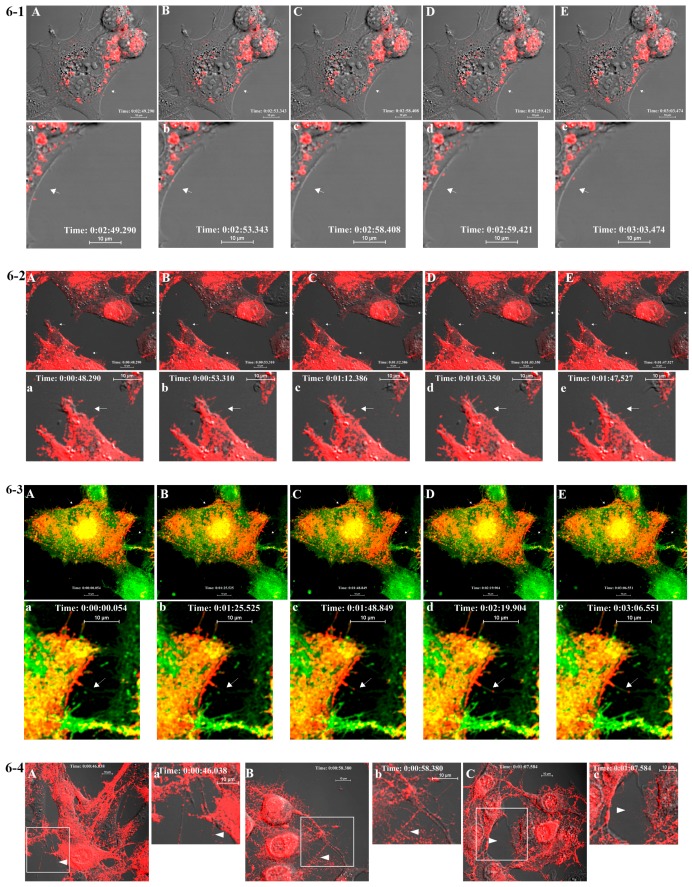
Live imaging of mCherry-E2 virus. (**6-1**) Time-lapse images of mCherry-E2 virus (panels **A**–**E**)-infected BHK cells exhibiting glycoprotein transport to the PM and virus budding at 3 h p.i. mCherry-E2 virus particles bud from the PM (white arrow) of infected cells. Selected images are shown from supplementary Video S1A (http://dx.doi.org/10.5281/zenodo.34119). Panels (**a**–**e**) indicate enlarged areas from panels (**A**–**E**) near the white arrow. Selected images are shown from supplementary Video S1B (http://dx.doi.org/10.5281/zenodo.34119); (**6-2**) Time-lapse images of mCherry-E2 virus budding from infected BHK cells (panels **A**–**E**) at 6 h p.i. Glycoprotein-containing vesicles traffic to the PM and virus buds from the PM. Budded viruses (white arrow) disperse from filopodial extensions to the surrounding media. Images correspond to Video S2A. Corresponding time-lapse images of enlarged area near the arrow are shown in panels (**a**–**e**) below, corresponding to supplementary Video S2B (http://dx.doi.org/10.5281/zenodo.34119). Arrow points to virus particle budding from infected cells traveling along the filopodial extension (Figure 6-2 panels **a**–**e**); (**6-3**) E2 glycoprotein (mCherry-E2; red) colocalizing with Golgi stain (panels **A**–**E**) observed in live imaging with images from supplementary Video S5A (http://dx.doi.org/10.5281/zenodo.34119). BHK cells were infected with mCherry-E2 virus and stained with BODIPY FL C5 ceramide at 3 h p.i. and imaged at 4 h p.i. Yellow color represents the colocalization of Golgi and mCherry-E2. Glycoprotein-containing (panel **B**) vesicles originate from Golgi and are transported to the PM. Enlarged areas of panels 6-3 **A**–**E** are shown in panels (**a**–**e**), and selected images are from Video S5B; (**6-4**) Virus particles budding predominantly from filopodial extensions present at the PM were dispersed into the media after treating cells with pH 7 (panels **A**,**B**) for 15 min at 6 h p.i. When cells were imaged after low pH (pH 5) treatment, budded virions that were outside the cell and on the filopodial extensions stayed attached to the filopodia outside the cells and fused to the PM of the filopodial extensions (panels **C** and **D**) while retaining red fluorescence (white arrow). When cells were treated with pH 4 (panels **E** and **F**), the fluorescence was lost from the virus particles that were fusing to the PM. However, the fluorescent signal from the mCherry-E2 protein molecules present within the cell was not lost after low pH treatment. Insets labeled **B**, **D**, and **F** represent enlarged rectangle areas from representative panels **A**, **C**, and **E** on the left.

BHK cells infected with WT or FP-tagged SINV were subjected to immunofluorescence (IF) analyses using antibodies against giantin, E2, E1, and CP (Figure 5). All structural proteins were located on the PM (Figure 5B,C,F) and glycoproteins E1 and E2 were detected on filopodial extensions in both WT and FP-tagged, virus-infected cells (Figure S1). The association of glycoproteins with the Golgi complex in virus-infected cells was studied using anti-Giantin antibody (Figure 5A,D). In BHK cells infected with mCherry-E2 virus, glycoprotein-containing vesicles (Figure 6-1) were transported to the PM (Videos S1A,B and S2A,B). Time-lapse images of mCherry-E2 virus-infected BHK cells showed virus budding in close association with filopodial extensions (Figure 6-2, Video S2A,B). Anterograde trafficking of glycoprotein-containing vesicles to the PM was observed in BHK cells where nascent virions were budding (Videos S1 and S2). Budded viruses were released from filopodial extensions to the surrounding media (Figure 6-2). BHK cells transfected with non-budding cdE2 mutant _416_CC_417_/AA (Video S3) show the lack of particle budding from filopodial extensions despite glycoprotein transport to the PM and filopodial extensions (Video S3).

BHK cells transfected with an mCherry-E2 tagged E1 fusion loop mutant (G91D) showed that particles released from transfected cells can enter uninfected neighboring cells, but they appear unable to fuse within the cell to initiate a productive infection (Video S4). The association of mCherry-E2 with Golgi was analyzed in live imaging using BODIPY FL C5 ceramide (Figure 6-3) which stains Golgi (Videos S5A and S5B). Additionally, virus budding in close association with filopodial extensions and fluorescent single-particle trafficking between two cells was observed (Figure 6-3 panels a–e and Video S5B) in the stained cells (green). Furthermore, low pH treatment of FP-tagged, virus-infected BHK cells confirmed that the budded virions were present outside the filopodial extensions. At neutral pH, viruses were present outside the PM of filopodial extensions and were released into the media (Figure 6-4 panels A and B). Low pH treatment of BHK cells infected with FP-tagged virus showed that the budded virions retained the fluorescence and stayed attached to the filopodia after fusing to the PM at pH 5 (Figure 6-4 panels C and D). When cells were treated at pH 4, the fluorescent signals were lost from the fused virions on the filopodia as well as the glycoprotein spikes present on the PM while the fluorescence was retained for the mCherry-E2 present within the cells (Figure 6-4 panels E and F).

### 3.4. TEM Analysis of BHK Cells Infected with WT-SINV

**Figure 7:**
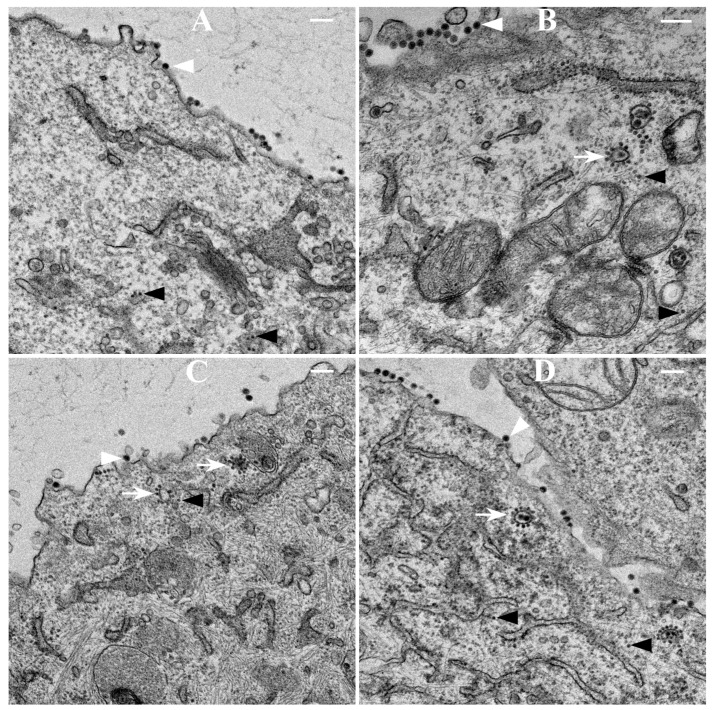
Transmission electron microscopy (TEM) analysis of SINV-infected BHK cells. BHK cells were infected with WT or mCherry-E2 virus at an MOI 5 and fixed for TEM analysis at 6 h and 12 h. p.i. Cells infected with WT (panel **A** 6 h; panel **B** 12 h) and cells infected with mCherry-E2 (Panel **C** 6 h; panel **D** 12 h) viruses are shown. Budding viruses (white arrowhead), and nucleocapsid cores (black arrows) are marked. White arrows indicate type II cytopathic vacuoles (CPV-II). Scale bars represent 200 nm.

Thin sections of BHK cells infected with WT SINV at 6 h p.i. (Figure 7A) and 12 h p.i. (Figure 7B) and mCherry-E2 SINV at 6 h p.i. (Figure 7C) and 12 h p.i. (Figure 7D) showed the presence of NCs, budding viruses, and released virions. CPV-IIs with NCs attached to the outer lipid bilayers were seen in the cytoplasm (Figure 7B,D). Virus budding occurred predominantly from the PM. When compared to 6 h p.i., CPV-IIs were abundant at 12 h p.i. (Figure 7B,D). Virus budding is associated with filopodial extensions and filopodia were observed in mCherry-E2 virus-infected BHK cells at 12 h p.i. (Figure S2). Overall, mCherry-E2 virus-infected cells were indistinguishable from WT SINV-infected cells. CPV-II and virus budding from the PM were observed from both types of virus-infected cells, suggesting that these processes were not affected by the presence of the fluorescent protein tag on the mCherry-E2 virus.

## 4. Discussion

Single-particle tracking and real-time live imaging provide powerful tools for obtaining spatial and temporal resolution information. This contrasts with traditional modes of TEM and super resolution light microscopy that provide high spatial resolution but lack temporal resolution. In this study, we used live imaging coupled with an FP-tagged viral protein to analyze temporal aspects of alphavirus assembly in mammalian cells. We generated an FP-tagged virus with mCherry fused to the N-terminus of the E2 glycoprotein, which is known to tolerate insertions of the immunoglobulin-binding domains of protein L [39] and fluorescent proteins [17,18,19,20]. In this study, mCherry was deemed to be an ideal tag based on its monomeric nature, photostability, fast maturation, and resistance to low pH [41]. The mCherry tag has a low pK_a_ value of 4.5, and hence retains its fluorescence when it encounters the cellular secretory pathway [42]. During maturation, E3 packs against the acid-sensitive region of E2, which maintains the A and B domains of E2 and the B domain to cover the E1 fusion loop, thus protecting the virus from premature fusion with other cellular membranes [40]. After furin cleaves E3, acidification of the virus during entry causes E2 domain B to move away from its neutral pH position and exposes the fusion loop [4]. We have demonstrated that the mCherry tag did not adversely affect any of these functions of E3 and E2.

We determined the 3D cryoEM structure of the mCherry-E2 virus to assess the effects, if any, of the 236-residue insertion on the structural integrity of the virus and its potential to alter the virus lifecycle. The cryo-reconstruction of the mCherry-E2 virus at 11 Å resolution revealed that the overall size of the virion and the icosahedral arrangement of the E1, E2, and CP proteins remained essentially unaffected despite the presence of the large FP insertion. The 240 copies of the mCherry tag wedge tightly between neighboring spikes, and this arrangement causes a slight rearrangement of the spikes as well as the nucleocapsid protein (NCP) pentamers and hexamers and small conformational changes in the membrane bilayer. This confirms our previous observation that minor conformational adjustments of the viral glycoprotein spikes get transmitted radially to the NC via the strong interactions that occur between the inner NC and the outer glycoprotein layers [6]. These small alterations, coupled with the delay in surface expression of the viral glycoproteins as demonstrated by flow cytometry analysis, contribute to the 10-fold reduction in mCherry-E2 virus growth. However, the presence of the mCherry tag did not affect the receptor binding or fusion functions of E2 and E1, respectively. The cryoEM structure of the FP-tagged SINV also confirmed that there is a 1:1 ratio of mCherry to E2 in every virion, which leads to a strong fluorescence signal and thus greatly facilitates single-particle tracking experiments.

We examined mCherry-E2 SINV in live cell imaging primarily to demonstrate virus assembly and budding in real-time using a FP-tagged virus that has been characterized as structurally stable. At times as early as 3 h p.i. we detected budding of FP-tagged virus particles from infected BHK cells, and we gleaned additional information about virus budding and dissemination by examining virus budding and entry mutants. By moving budding viral particles away from the PM of infected cells, the filopodia may act to suppress superinfection, possibly by reducing the re-attachment into the infected cells. As the first step to probe SINV entry and fusion in late endosomes and to study the mechanism of virus fusion, we generated a G91D fusion loop mutation in E1, which abrogates low-pH-triggered fusion and infection [43]. The mCherry-E2 with the G91D fusion loop mutation in E1 was released from the transfected cells, but was unable to fuse and became trapped presumably in the endosome after entry. Using cdE2-NC interaction-deficient, non-budding mCherry-E2 mutants _400_YAL_402_/A3 and _416_CC_417_/A2 we show that non-budding, FP-tagged cdE2 mutations are sufficient to stop fluorescent particle budding from transfected cells. Additionally, using live imaging, we describe that virus budding occurs at the PM for both wild-type and mCherry-E2 virus by the interaction of surface glycoproteins that are transported to the PM via cytopathic vacuoles. We characterized these cytopathic vacuoles using TEM, and live imaging has shown that they contain E2 glycoproteins on their membranes. NCs were also found on the outer membrane of these vacuoles by TEM analysis. As the virus assembly sites are established on the PM, the budded virions utilize filopodial extensions for spreading away from the infected cells.

Similar to the WT SINV, in the mCherry-E2 virus construct, the furin cleavage occurs after the E3 coding sequence, but before mCherry-E2. Data from our virus characterization and imaging experiments of the mCherry-E2 virus suggest that the presence of mCherry after the furin cleavage site on pE2 does not cause significant virus assembly and entry defects. While the E3 protein is cleaved in the Golgi from pE2 to yield the mature E2 protein, E3 stays associated with Venezuelan equine encephalitic virus (VEEV) even after furin cleavage, as evidenced from the cryoEM structure of mature VEEV [44]. Although, in this structure, densities could be attributed to the two alpha-helices of E3, due to disconnected densities, a high resolution E3 density map was not obtained for the cryoEM map of mature VEEV containing cleaved E3. Nevertheless, the observed E3 density decorating the outermost portion of E2 above subdomains A and B was similar to the position of E3 in the pE2 cleavage-impaired, immature SINV mutant virus [45]. These observations have suggested that E3 functions to maintain the relative orientation between E2 subdomains A and B, so as to protect the E1 fusion loop from premature exposure to the host membranes [4,40]. However, E3 does not stay associated with mature SINV after cleavage [6,40]. In our FP-tagged virus, the mCherry density is buried between neighboring glycoprotein spikes and does not occupy the position of E3 over the E2 acid-sensitive region. We hypothesize that this property of the FP-tagged virus is possibly because of the flexible linker region between E3 and E2 (between E3 and mCherry in the FP-tagged virus) that allows sufficient movement of E3 to still maintain its position on E2 to protect the acid-sensitive region of E2 during glycoprotein maturation of the mCherry-E2 virus. Thus, our cryoEM structure explains the unusual stability of the FP-tagged virus.

The density map of the mCherry-E2 virus reveals strong density extending from the N-terminus of E2 which is absent in the wild-type virus (Figure 4C). This density of mCherry can be seen near the five-fold axis between adjacent spikes in a close-up surface view of the virus. The shape and volume of the extra density closely fit the red fluorescent protein (RFP) crystal structure of a dimer but come from two different adjacent E2 molecules (Figure 4D) from two different spikes. Additionally, mCherry appears to make several contacts with the glycoprotein spikes, possibly adding to the stability of the tag. Similar observations were reported for a cryoEM density map of HSV-1 with a GFP-labeled UL17 capsid protein where the freedom of movement of the GFP tag was restricted due to the contact between the GFP tag and capsid density that was sufficient to prevent delocalization of the tag density but without abrogating formation of the capsid vertex-specific component heterodimer [26]. The mCherry tag thus gives additional stability for the FP-tagged virus and explains the accommodation of 240 copies of the mCherry molecule without increasing the diameter of the particles.

Correlative light and electron microscopy (CLEM) studies using fluorescently tagged SINV have indicated the importance of filopodial extensions as preferred sites for alphavirus production, and they appear to mediate cell-cell virus particle transfer [18]. By live imaging, Martinez *et al.* have shown that long cellular extensions are involved in alphavirus cell-to-cell particle transfer [18]. Importantly, using fluorescent SINV virions, we demonstrate single-particle budding that spread from infected cells via filopodial extensions. Such virus budding was absent from cells transfected with RNA from an FP-tagged, cdE2 budding mutant. Using live imaging experiments that utilize FP-tagged viruses, we demonstrate that, in infected BHK cells, fluorescent vesicles containing glycoproteins are transported to the PM. These vesicles presumably originate from late Golgi and we provide evidence for the association of glycoprotein E2 with Golgi using a live imaging-compatible Golgi stain.

In mammalian cells, the viral glycoproteins reside on the membranes of ER, Golgi, CPV-II, and PM, and the virus eventually buds from the PM. We showed that the released FP-tagged virus could be immobilized onto the PM of the infected cells by low pH-mediated fusion at pH 5, confirming that the virus particles are outside the cell. The budded virions that fuse to the PM lost their fluorescence when the cells were treated at pH 4, which is below the pK_a_ of mCherry. Along with the fused virions, fluorescent glycoprotein spikes present on the PM also lost their fluorescence whereas the mCherry-E2 molecules inside the infected cells were protected from the low pH 4 treatment of the cell. Consistent with our previous findings [5,15], we show that the interaction of NC with the cell-surface glycoproteins generates virions that get propelled by the filopodial extensions, and we hypothesize that this process facilitates viral dissemination while preventing superinfection. SINV attachment factors such as heparan sulfate [46] and entry receptors such as NRAMP (divalent metal, ion transporter natural resistance-associated macrophage protein) [47] have been shown to enhance viral infection. These host factors and the mechanism of receptor-mediated endocytosis can be further investigated with this FP-tagged virus. Exploiting the FP-tagged mutant viruses generated in this study in conjunction with live imaging-compatible stains and labeled host proteins, high-resolution live imaging studies are ongoing with an aim to understand the various molecular interactions between viral glycoproteins and host proteins that are required for productive alphavirus receptor binding, entry, and fusion. Such high-resolution live imaging studies will provide new spatial and temporal information regarding various steps in the alphavirus lifecycle. 

## Supplementary Files

Supplementary File 1
