# Supplementary material for: Fluorescent Protein-Tagged Sindbis Virus E2 Glycoprotein Allows Single Particle Analysis of Virus Budding from Live Cells
Source: Viruses. 2015 Nov 27;7(12):6182–99. doi: 10.3390/v7122926 (PMC4690852; doi:10.3390/v7122926)
Supplement: Supplementary File 1 [file viruses-07-02926-s001.docx]

**Supplementary Information**

**
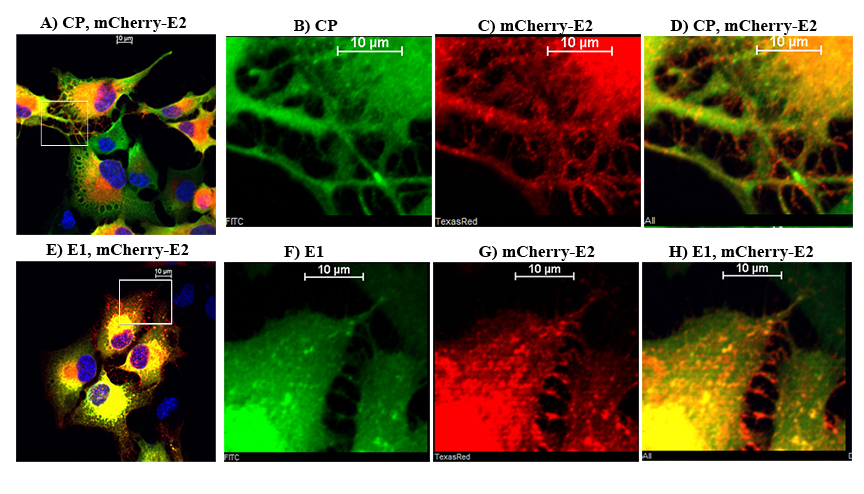
**

**Figure S1.** Enlarged view of panels E and F from figure 5 showing distribution of structural proteins on filopodial extensions. IF analysis mCherry-E2 virus infected BHK at 6 h p.i. with antibodies against rabbit polyclonal anti-CP (panels A-D) or rabbit polyclonal anti-E1 primary antibodies (panels E-H) and labeled with FITC-labeled goat anti-rabbit secondary antibody.


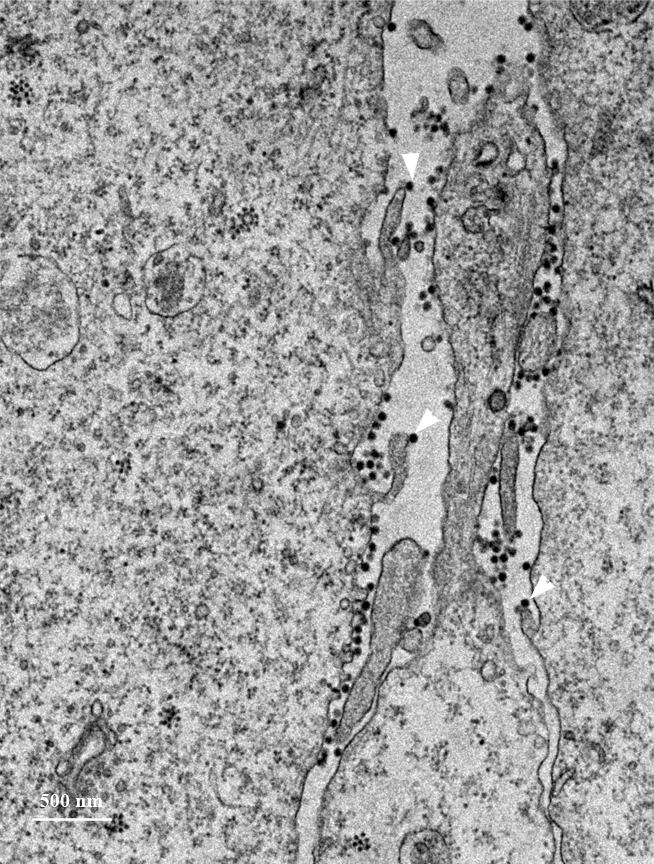


**Figure S2.** TEM analysis of mCherry-E2 virus infected BHK cells.BHK cells were infected with mCherry-E2 virus at an MOI 5 and fixed for TEM analysis at 12 h. p.i. White arrowhead represents a filopodial extension with budded virus particle on the outer surface of the PM. Scale bar represents 500 nm.

**Supplementary video legends**

**Video S1A.** (<http://dx.doi.org/10.5281/zenodo.34119>). BHK cells infected with mCherry-E2 virus at 3 h p.i. Glycoprotein containing vesicles are transported to the PM from where individual virions bud out. White arrow point to budding virions. Overall amount of glycoproteins present on the PM and the number of virus particles budding out are relatively reduced compared to the late stage of infection. Images were acquired at a rate of 0.99 fps and 75 frames were acquired. Video was generated using these images and played at a rate of 5 fps. Image acquisition time is shown as Time: hour: minute: second: millisecond (h:min:sec:msec ) and the scale bar represents 10 μm.

**Video S1B.** (<http://dx.doi.org/10.5281/zenodo.34119>). Enlarged area of video S1A showing budding virus particles from PM. White arrow indicates single particle post-budding moving away from the cell. Images were acquired at a rate of 0.99 fps and 75 frames were acquired. Video was generated using these images and played at a rate of 5 fps. Image acquisition time is shown as Time: hour: minute: second: millisecond (h:min:sec:msec ) and the scale bar represents 10 μm.

**Video S2A.** (<http://dx.doi.org/10.5281/zenodo.34119>). Virus budding and single particle movement associated with filopodial extensions observed from mCherry-E2 virus-infected BHK cells at 6 h p.i. Glycoprotein containing vesicle transport to the PM is also observed. Budded virions travel along the periphery of filopodia and are released from filopodial extensions to the surrounding media. Image acquisition was at a rate of 1 fps and 285 frames were acquired. Movie was generated using these images and played at a rate of 7 fps. Image acquisition time is shown as Time: h:min:sec:msec and the scale bar represents 10 μm.

**Video S2B.** (<http://dx.doi.org/10.5281/zenodo.34119>). Enlarged area of video S2A showing budding virus particles from filopodia. White arrow indicates virus budding from filopodial extensions. Images were acquired at a rate of 1 fps and the acquired 285 frames were used to generate the video at a rate of 7 fps. Image acquisition time is shown as Time: h:min:sec:msec and the scale bar represents 10 μm.

**Video S3.** (<http://dx.doi.org/10.5281/zenodo.34119>). BHK cells transfected with RNA from a non-budding cdE2 mutant _416_CC_417_/A2 mCherry-E2 virus. This non-budding mutant is unable to release fluorescent virus particles from the infected cells. The video shows the absence of fluorescent virus particle budding from the PM at 6 h post transfection even though the PM and filopodial extensions contain mCherry-E2. Despite the transport of glycoproteins to the PM, no fluorescent particles were released into the media. Yellow arrows point toward filopodial extensions. For the video, 304 images were acquired at a rate of 0.98 fps and the video was generated using the acquired images at a rate of 7 fps. Image acquisition time is shown as Time: h:min:sec:msec and the scale bar represents 10 μm.

**Video S4.** (<http://dx.doi.org/10.5281/zenodo.34119>). BHK cells transfected with RNA from an E1 Fusion loop (G91D) mutant of mCherry-E2 virus at 6 h post transfection. This non-fusing mutant produces fluorescent virus particles at a slower rate compared to WT that are unable to fuse after entering a new cell. White arrow points to fluorescent particles that are releasing into the media from filopodial extensions. Yellow arrow represents a fluorescent particle that had entered an adjacent un-transfected cell. A total of 149 images were acquired at a rate of 0.98 fps. Video was generated using these images at a rate of 7 fps. Image acquisition time is shown as Time: h:min:sec:msec and the scale bar represents 10 μm.

**Video S5A.** (http://dx.doi.org/10.5281/zenodo.34119). Glycoprotein E2 (mCherry-E2; red) colocalizing with Golgi stain (green) in BHK cells infected with mCherry-E2 virus and stained with BODIPY FL C5 ceramide at 5 h p.i. and imaged at 6 h p.i. Glycoprotein-containing red vesicles originate from Golgi as evidenced from the colocalization of red and green and these vesicles display anterograde transport to the PM and the virus particles are released by budding from the PM. Fluorescent particles are also seen budding from filopodial extensions (white arrows). Images were acquired at a rate of 0.13 fps for 295 seconds. Video was generated using these acquired images at a rate of 5 fps. Image acquisition time is shown as Time: h:min:sec:msec and the scale bar represents 10 μm.

**Video S5B.** (http://dx.doi.org/10.5281/zenodo.34119). An enlarged area of the video S5A near the white arrow showing movement of particles on filopodial extensions between two cells. Movie was played
at a rate of 5 fps. Image acquisition time is shown as Time: h:min:sec:msec and the scale bar represents 10 μm.

© 2015 by the authors; licensee MDPI, Basel, Switzerland. This article is an open access article distributed under the terms and conditions of the Creative Commons by Attribution (CC-BY) license (http://creativecommons.org/licenses/by/4.0/).
